# ﻿Five new species of the *Macrolycusligulatus* species-group from China (Coleoptera, Lycidae)

**DOI:** 10.3897/zookeys.1208.125938

**Published:** 2024-08-01

**Authors:** Ruolan Du, Yuxia Yang, Xingke Yang, Haoyu Liu

**Affiliations:** 1 Key Laboratory of Zoological Systematics and Application, School of Life Sciences, Hebei University, Baoding 071002, China; 2 Hebei Basic Science Center for Biotic Interaction, Hebei University, Baoding 071002, China; 3 Key Laboratory of Zoological Systematics and Evolution, Institute of Zoology, Chinese Academy of Sciences, Beijing 100101, China

**Keywords:** Alpha taxonomy, China, Macrolycus, Net-winged beetles, new species

## Abstract

Five new species of the *Macrolycusligulatus* species-group, *M.expansus***sp. nov.**, *M.quartus***sp. nov.**, *M.costus***sp. nov.**, *M.opacipennis***sp. nov.** and *M.curtus***sp. nov.**, are reported from China and described with macrophotographs of the habitus of both sexes and aedeagi. *Macrolycusguangxiensis* Li, Bocak & Pang, 2015 is illustrated showing the female habitus and genitalia for the first time. In addition, a distribution map and a key to all species of the *M.ligulatus* species-group are provided.

## ﻿Introduction

Net-winged beetles of the genus *Macrolycus* Waterhouse, 1878 sensu lato are widely distributed in the Oriental and eastern Palaearctic regions (Nakane 1969, 1994; Kazantsev 1993, 2001, 2002, 2013; Li et al. 2012, 2015; Liu et al. 2023; Du et al. 2024). It is the sole member of the tribe Macrolycini, currently placed in the subfamily Ateliinae Kleine, 1928 of Lycidae (Kusy et al. 2019). A total of 73 *Macrolycus* species have been recorded until now (Liu et al. 2023; Du et al. 2024), divided into nine species-groups based on molecular phylogeny (Li et al. 2015). Among them, the *M.ligulatus* species-group can be distinguished from others by the phallus usually expanded ventrodistally, and present with a U- or V-shaped notch and a tongue-like lamella at the apex (Li et al. 2015).

Eight species are currently included in the *M.ligulatus* species-group (Li et al. 2015). Recently, we assembled a large series of *Macrolycus* material from China and discovered dozens of new species that are currently being described or will be described based on their respective groups (Li et al. 2015). In this study, our focus lies on the *M.ligulatus* species-group, and we present five new species below.

## ﻿Material and methods

The studied material is preserved in the Institute of Zoology, Chinese Academy of Sciences, Beijing, China (**IZAS**) and the Museum of Hebei University, Baoding, China (**MHBU**). We identified the species based on the works of Kazantsev (1993, 2001, 2002, 2013), Li et al. (2012, 2015) and Li (2015). The description format follows Li et al. (2012, 2015), and the terminology of female genitalia follows Kazantsev (2005).

The specimens were first softened in water, and then the genitalia of both sexes were dissected. After dissection, the male genitalia was cleared in 10% NaOH solution, examined and photographed in glycerol, and finally glued on a paper card for permanent preservation. The female genitalia was dyed with hematoxylin, examined in 75% alcohol and preserved in glycerol. Images of the adults were taken with a Canon EOS 80D digital camera and those of the genitalia by a Leica M205A stereomicroscope, which were stacked in Helicon Focus ver. 7. The final plates were edited in Adobe Photoshop CS3.10.0.1.

The measurements were taken with Image J ver. 1.50i (NIH, Bethesda, MD, USA). Body length was measured from the anterior margin of the head to the elytral apex, and the width across the elytral humeri. Pronotal length was measured from the middle of the anterior margin to the middle of the posterior margin of the pronotum, and the width across its widest part. Eye diameter was measured at the maximal width and the interocular distance at the minimal point. The length of the lamella of the antennae was measured from the apex to the middle of the joint itself. The aedeagus was measured and compared at the maximal width of the basal part, subapical part and apex in ventral views, respectively.

The distribution information was collected from the literature (Kazantsev 1993, 2001, 2013; Li et al. 2012, 2015; Li 2015) and the present studied material. The distribution map was prepared using ArcMap ver. 10.8 and edited in Photoshop CS3.10.0.1.

## ﻿Results

**Class Insecta Linnaeus, 1758**

**Order Coleoptera Linnaeus, 1758**

**Family Lycidae Laporte, 1836**

**Tribe Macrolycini Kleine, 1933**

**Genus *Macrolycus* Waterhouse, 1878**

### ﻿*Macrolycusligulatus* species-group

**Figure 1.**
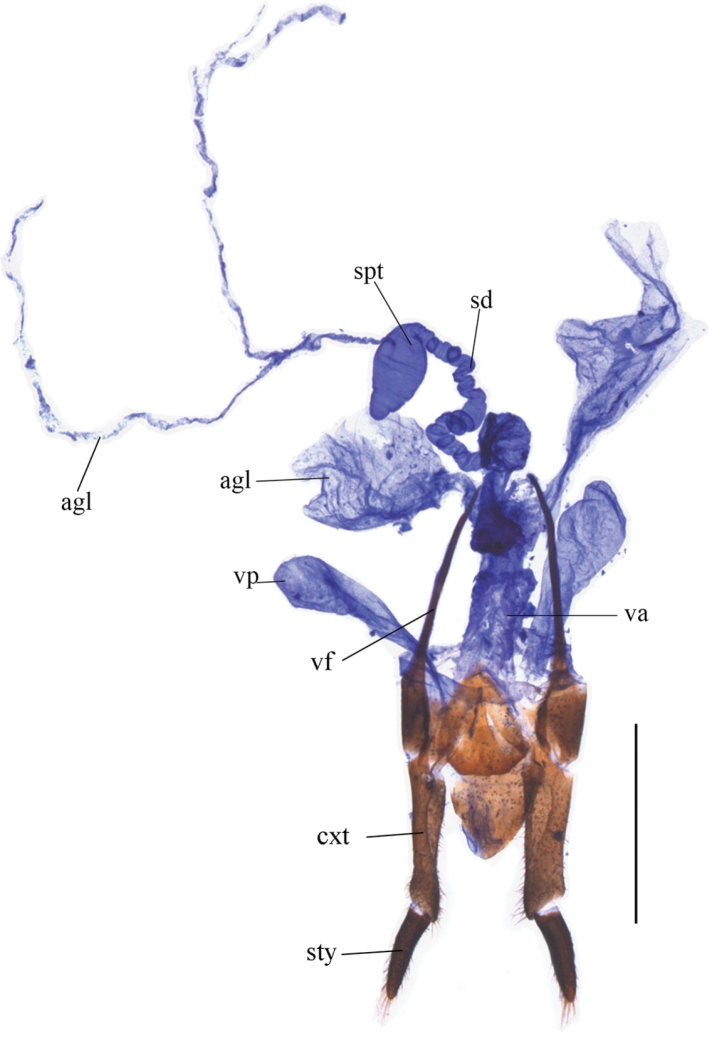
Internal organ of female reproductive system (ventral view) of *Macrolycusguangxiensis* Li, Bocak & Pang, 2015. Scale bars: 1.0 mm. Abbreviations: agl–accessory gland; va–vagina; vp–vaginal pouch; spt–spermatheca; sd–seminal duct; vf–valvifer; cxt– coxite; sty–stylus.

**Updated diagnosis.** Female external genitalia (Fig. 1): valvifers free, styli slender and cylindrical, coxites elongate. Internal organ of female reproductive system (Fig. 1): vagina elongate and globular at apex, present with a pair of large vaginal pouches on both sides of the basal part and a pair of accessory glands, which are greatly expanded distad and separated on both sides of the apical part; seminal duct spiral tube-shaped; spermatheca rugby-shaped, present with a thin and bifurcate accessory gland arising from middle part. Male genitalia with phallus usually with a U- or V-shaped notch and a ventrally inclined and tapered or square lamella at apex (e.g., Figs 4, 6).

**Included species.***Macrolycusnotaticollis* Pic, 1935, *M.praecellens* Kazantsev, 1993, *M.bocakorum* Kazantsev, 2001, *M.extrusus* Li, Bocak & Pang, 2012, *M.ligulatus* Li, Bocak & Pang, 2012, *M.chapaensis* Kazantsev, 2013, *M.guangxiensis* Li, Bocak & Pang, 2015, *M.parvus* Li, Bocak & Pang, 2015, *M.expansus* sp. nov., *M.quartus* sp. nov., *M.costus* sp. nov., *M.opacipennis* sp. nov. and *M.curtus* sp. nov.

**Figure 2.**
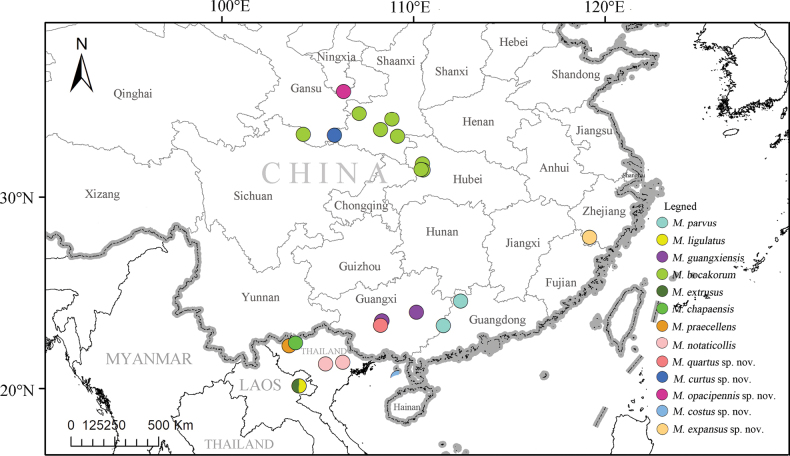
Distribution map of the *Macrolycusligulatus* species-group in the world.

**Distribution (Fig. 2).** China (Ningxia, Gansu, Shaanxi, Sichuan, Guizhou, Zhejiang, Guangxi, Guangdong), Laos, Vietnam.

**Remarks.** The female reproductive system of *Macrolycus*, encompassing external genitalia and internal organs, with *M.guangxiensis* as the representative species, is presented here for the first time.

#### ﻿ Macrolycus guangxiensis

Taxon classificationAnimaliaColeopteraLycidae

Li, Bocak & Pang, 2015

D0FBB45F-14A1-5693-8A28-34A400C2D5AA

Figs 3A , 4A–C

Macrolycus guangxiensis Li, Bocak & Pang, 2015: 326, figs 9, 28, 29, 45, 55.

##### Material examined.

China: 5♂8♀ (MHBU), Guangxi, Wuming, Damingshan, 1230– 1423 m, 20.v.2011, leg. H. Y. Liu.

##### Descriptive notes.

**Male.** Phallus (Fig. 4A–C) slender, nearly parallel-sided at basal part in dorsal and ventral views (Fig. 4A, B), subapical part moderately and asymmetrically inflated laterally, about 1.45 times as wide as basal part, with an oval ventral-cavity, apical part progressively constricted distad, apex with a deep V-shaped notch, about 0.19 times as wide as subapical part; basal 1/3 part curved ventrally in lateral view (Fig. 4C), subapical part feebly inflated ventrally, apical part moderately expanded ventrally, and apex with a tapered lamella.

**Figure 3.**
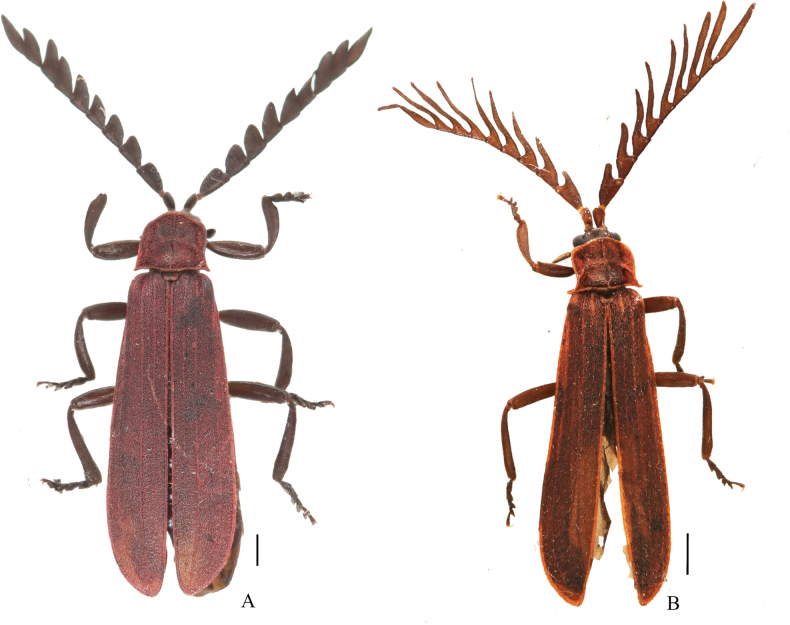
Habitus, dorsal views of *Macrolycusguangxiensis* Li, Bocak & Pang, 2015 (**A**) and *M.expansus* sp. nov (**B**). **A** female **B** male. Scale bars: 1.0 mm.

**Female** (Fig. 3A). Similar to males, but larger in body size. Length 11.8–13.2 mm, width at humeri 2.5–2.8 mm. Antennae serrate and antennomeres III–X long- or wide-triangular. Pronotum 1.2 times wider than long, anterior angles obtuse-angled. Elytra 3.6 times longer than humeral width.

**Figure 4.**
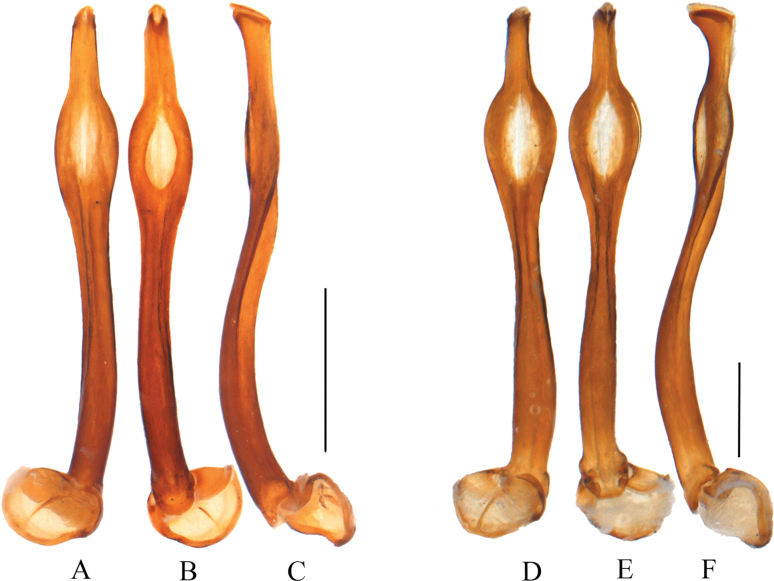
Aedeagi of *Macrolycusguangxiensis* Li, Bocak & Pang, 2015 (**A–C**) and *M.expansus* sp. nov. (**D–F**) **A, D** dorsal views **B, E** ventral views **C, F** lateral views. Scale bars: 1.0 mm.

##### Distribution

(Fig. 2). China (Guangxi).

##### Remarks.

We provide an illustration of the female habitus and a comparison with the males of this species for the first time.

#### ﻿ Macrolycus expansus

Taxon classificationAnimaliaColeopteraLycidae

Y. Yang, Liu & X. Yang sp. nov.

6E335F7E-9B89-5321-97C1-A210C748910C

https://zoobank.org/9C3D89C2-894A-4C16-BAF9-831BD49827FD

Figs 3B , 4D–F

##### Diagnosis.

The species resembles *M.guangxiensis* Li, Bocak & Pang, 2015 in the general shape of the phallus, but differs in the longer lamella of male antennomere III, 0.9 times as long as the joint itself (Fig. 3B); phallus with subapical part strongly inflated laterally in ventral view (Fig. 4E), apical part strongly expanded ventrally in lateral view (Fig. 4F). In comparison, in *M.guangxiensis* the lamella of male antennomere III is shorter and 0.7 times as long as the joint itself (Li et al. 2015: fig. 45), the subapical part of phallus is moderately inflated laterally in ventral view (Fig. 4B), and the apical part is moderately expanded ventrally in lateral view (Fig. 4C).

##### Etymology.

The specific name is derived from the Latin *expansus* (to expand), referring to its strongly expanded subapical part of the phallus.

##### Type material.

***Holotype*.** China: ♂ (IZAS), Zhejiang, Longquan, Fengyangshan, 29.vii.2007, leg. L. K Tan.

##### Description.

**Male** (Fig. 3B). Length 9.3 mm, width at humeri 1.9 mm.

Body black. Pronotum, elytra and scutellum dark red. Surface covered with decumbent red pubescence (Fig. 3B).

Eyes small, interocular distance about 1.6 times greater than eye diameter. Antennae flabellate, overlapping basal 2/3 length of elytra when inclined. Antennomeres III–XI lamellate; lamella of III 0.9 times as long as joint itself and rounded apically; lamellae of IV–XI pointed at apices, lamella of IX longest, 3.9 times longer than joint itself (Fig. 3B).

Pronotum square, 1.2 times wider than long. Anterior margin widely rounded and projecting anteriad, lateral margins feebly sinuate and posterior margin bisinuate; anterior angles confluent with anterior margin, posterior angles sharp and moderately projected. Scutellum trapezoidal, feebly emarginate at apex (Fig. 3B).

Elytra 3.9 times longer than humeral width. Costae I and III weak but visible along its length, IV as strong as II (Fig. 3B).

Phallus slender (Fig. 4D–F), basal part stout and distinctly narrowed towards middle in dorsal and ventral views (Fig. 4D, E), subapical part strongly and asymmetrically inflated laterally, about 2.8 times as wide as basal part, with an oval ventral-cavity, apical part nearly parallel-sided, apex with a deep V-shaped notch, about 0.19 times as wide as subapical part; basal 1/3 part stout and curved ventrally in lateral view (Fig. 4F), subapical part moderately inflated ventrally, apical part distinctly expanded ventrally, with a tapered lamella.

##### Distribution

**(Fig. 2).** China (Zhejiang).

#### ﻿ Macrolycus quartus

Taxon classificationAnimaliaColeopteraLycidae

Y. Yang, Du & Liu sp. nov.

F00F8690-CB1F-5416-B2B7-D12284C81814

https://zoobank.org/809EBD56-644B-416A-B944-6A8E12A3F738

Figs 5A, B , 6A–C

##### Diagnosis.

**Figure 5.**
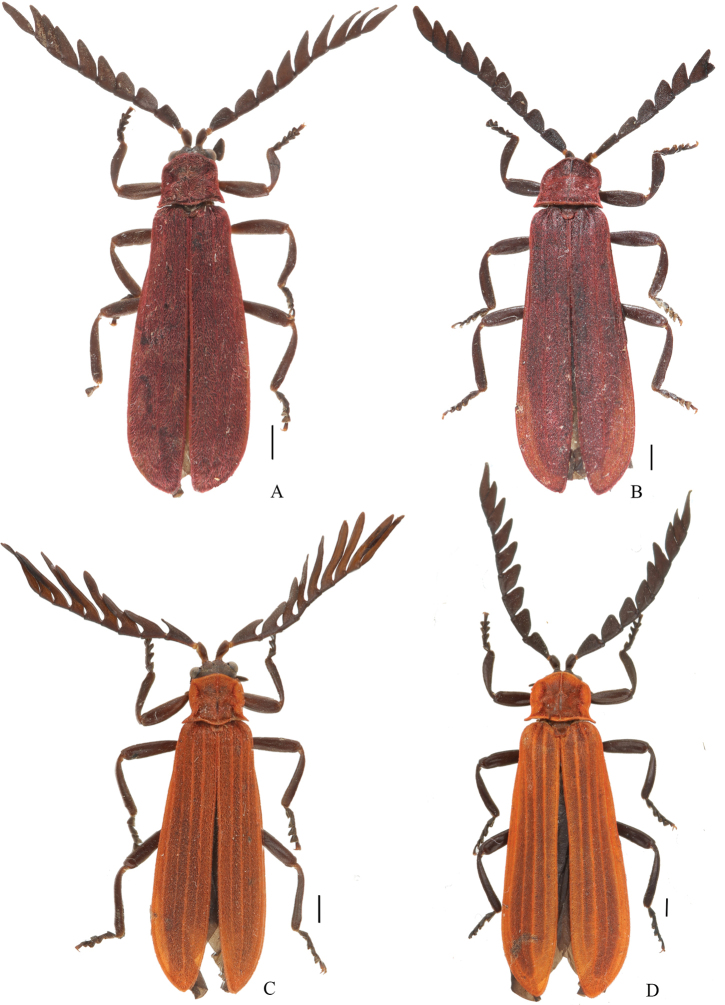
Habitus (dorsal views) of *Macrolycusquartus* sp. nov. (**A, B**) and *M.costus* sp. nov. (**C, D**) **A, C** males **B, D** females. Scale bars: 1.0 mm.

**Figure 6.**
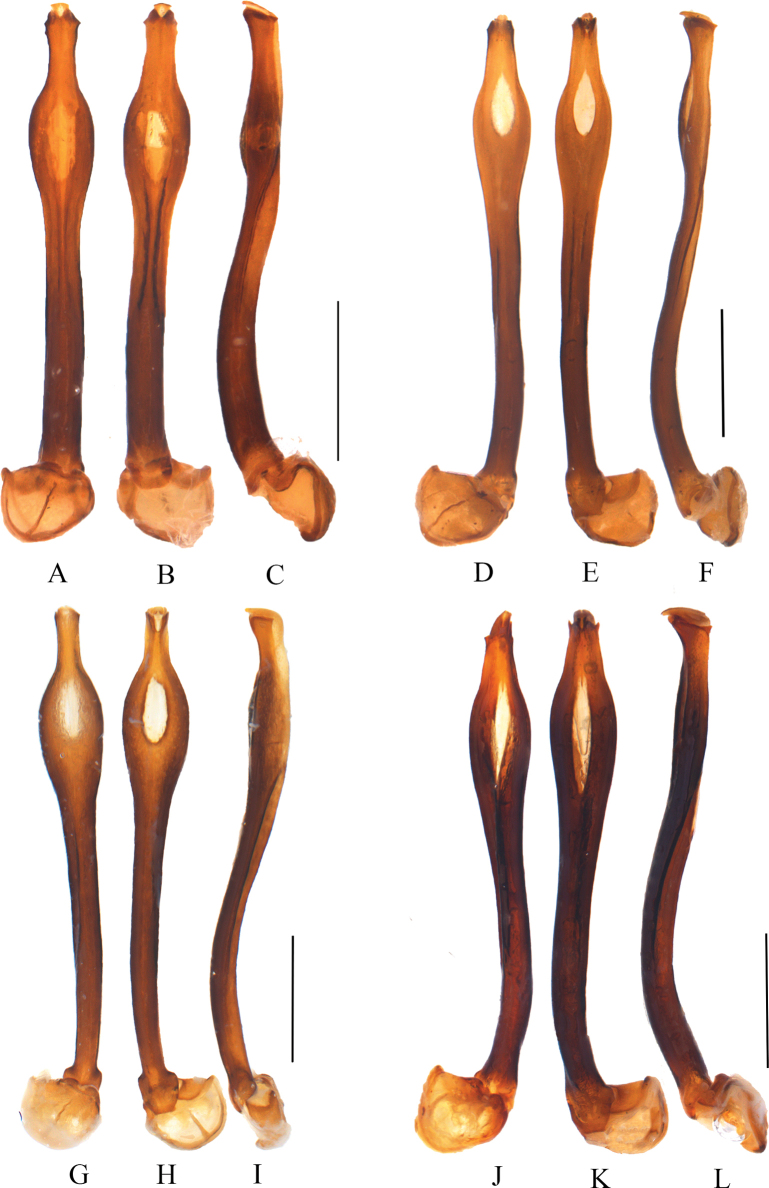
Aedeagi of *Macrolycusquartus* sp. nov. (**A–C**), *M.costus* sp. nov. (**D–F**), *M.opacipennis* sp. nov. (**G–I**) and *M.curtus* sp. nov. (**J–L**) **A, D, G, J** dorsal views **B, E, H, K** ventral views **C, F, I, L** lateral views. Scale bars: 1.0 mm.

The species resembles *M.praecellens* Kazantsev, 1993, but can be distinguished from the latter by the following characters: lamellae of antennomeres III and IV obtuse at apices (Fig. 5A); phallus integrally stout (Fig. 6A–C), apical part relatively long and moderately expanded ventrally in lateral view (Fig. 6C). In contrast, in *M.praecellens*, lamellae of antennomeres III and IV are acute at apices (Kazantsev 1993: fig. 13); phallus is integrally slender, apical part is relatively short and strongly expanded ventrally in lateral view (Kazantsev 1993: fig. 12).

##### Etymology.

The specific name is derived from the Latin *quartus* (the fourth), referring to its antennomere IV truncated at apex.

##### Type material.

***Holotype*.** China: ♂ (MHBU), Guangxi, Wuming, Damingshan, 1100 m, 27.v.2011, leg. H. Y. Liu. ***Paratypes*.** 3♂9♀ (MHBU), same data as the holotype; 3♂7♀ (MHBU), Guangxi, Wuming, Damingshan, 1230–1422 m, 20.v.2011, leg. H. Y. Liu.

##### Description.

**Male** (Fig. 5A). Length 9.4–10.6 mm (10.0 mm in holotype), width at humeri 1.9–2.3 mm (2.2 mm in holotype).

Body black. Pronotum, elytra and scutellum dark red. Surface covered with decumbent red pubescence (Fig. 5A).

Eyes small, interocular distance about 2.3 times greater than eye diameter. Antennae flabellate, overlapping basal 2/3 length of elytra when inclined. Antennomere III triangular and obtuse apically, about 2.5 times longer than wide; IV–XI lamellate, lamella of IV apically obtuse and lamellae of V–XI pointed at apices; lamella of IX longest, 3.8 times longer than joint itself (Fig. 5A).

Pronotum square, 1.1 times wider than long. Anterior margin projecting anteriad and feebly emarginate at apex, lateral margins sinuate and posterior margin bisinuate; anterior angles rounded, posterior angles sharp and sharply projected. Scutellum trapezoidal, feebly emarginate at apex (Fig. 5A).

Elytra 3.8 times longer than humeral width. Costa I weak, II as strong as IV, and III weak and visible only at humeri (Fig. 5A).

Phallus slender (Fig. 6A–C), nearly parallel-sided basally in dorsal and ventral views (Fig. 6A, B), subapical part moderately and asymmetrically inflated laterally, about 1.89 times as wide as basal part, with an oval ventral-cavity, apical part progressively expanded distad, apex with a shallow V-shaped notch, about 0.54 times as wide as subapical part; basal 1/3 part feebly curved ventrally in lateral view (Fig. 6C), subapical part inflated ventrally, apical part moderately expanded ventrally, with a square lamella.

**Female** (Fig. 5B). Similar to male, but larger in body size. Length 11.5–13.9 mm, width at humeri 2.7–3.3 mm. Antennae serrate and shorter, overlapping elytral mid-length when inclined. Pronotum 1.2 times wider than long, anterior angles obtuse-angled.

##### Distribution

**(Fig. 2).** China (Guangxi).

#### ﻿ Macrolycus costus

Taxon classificationAnimaliaColeopteraLycidae

Y. Yang, Du & Liu sp. nov.

A9B4FA5F-79D1-51F9-897C-E3AD74EE9A2C

https://zoobank.org/C4DA9323-4AE2-4625-872F-9FEC27E2B12E

Figs 5C, D , 6D–F

##### Diagnosis.

The species resembles *M.guangxiensis*, but differs in the male antennae overlapping basal 2/3 length of elytra when inclined and a strong elytral costa III (Fig. 5C), phallus extremely slender at basal part in ventral view (Fig. 6D). Unlike in *M.guangxiensis*, the male antennae only reach elytral mid-length, elytral costa III is usually weak (Li et al. 2015: fig. 9), and phallus is relatively stout basally in ventral view (Li et al. 2015: fig. 29).

##### Etymology.

The specific name is derived from the Latin *costa* (a rib), referring to its strong elytral costae III.

##### Type material.

***Holotype*.** China: ♂ (MHBU), Guangxi, Wuming, Damingshan, 1100 m, 27.v. 2011, leg. H. Y. Liu. ***Paratypes*.** 3♂4♀ (MHBU), same data as the holotype.

##### Description.

**Male** (Fig. 5C). Length 12.4 mm, width at humeri 2.8 mm.

Body black. Pronotum, elytra and scutellum red. Costae of elytra orange red. Surface covered with decumbent red pubescence (Fig. 5C).

Eyes small, interocular distance about 1.6 times greater than eye diameter. Antennae flabellate, overlapping basal 2/3 length of elytra when inclined. Antennomeres III–XI lamellate, lamellae pointed at apices; lamella of III 0.6 times as long as joint itself; lamella of VIII longest, 4.2 times longer than joint itself (Fig. 5C).

Pronotum square, 1.1 times wider than long. Anterior margin widely rounded, lateral margins strongly sinuate and posterior margin bisinuate; anterior angles obtuse-angled, posterior angles sharp and moderately projected. Scutellum trapezoidal, straight at apex (Fig. 5C).

Elytra 3.6 times longer than humeral width. Costa I as strong as II, III and IV (Fig. 5C).

Phallus slender (Fig. 6D–F), basal part parallel-sided in dorsal and ventral views (Fig. 6D, E), subapical part strongly and asymmetrically inflated laterally, about twice as wide as basal part, with a fusiform ventral-cavity, apical part progressively narrowed distad, apex with a shallow V-shaped notch, about 0.46 times as wide as subapical part; basal 1/4 part curved ventrally in lateral view (Fig. 6F), subapical part moderately inflated ventrally, apical part moderately expanded ventrally, and with a tapered lamella.

**Female** (Fig. 6D). Similar to males, but larger in body size. Length 17.0–18.2 mm, width at humeri 4.0–4.5 mm. Antennae serrate and shorter, overlapping elytral mid-length when inclined. Pronotum 1.3 times wider than long, anterior angles obtuse-angled. Elytra 3.4 times longer than humeral width.

##### Distribution

**(Fig. 2).** China (Guangxi).

#### ﻿ Macrolycus opacipennis

Taxon classificationAnimaliaColeopteraLycidae

Y. Yang, Du & Liu sp. nov.

8C86DBAB-936D-5296-BAD2-F8A9882D7798

https://zoobank.org/559D7E0F-0505-4958-A098-D8D381CD8480

Figs 6G–I , 7A, B

##### Diagnosis.

**Figure 7.**
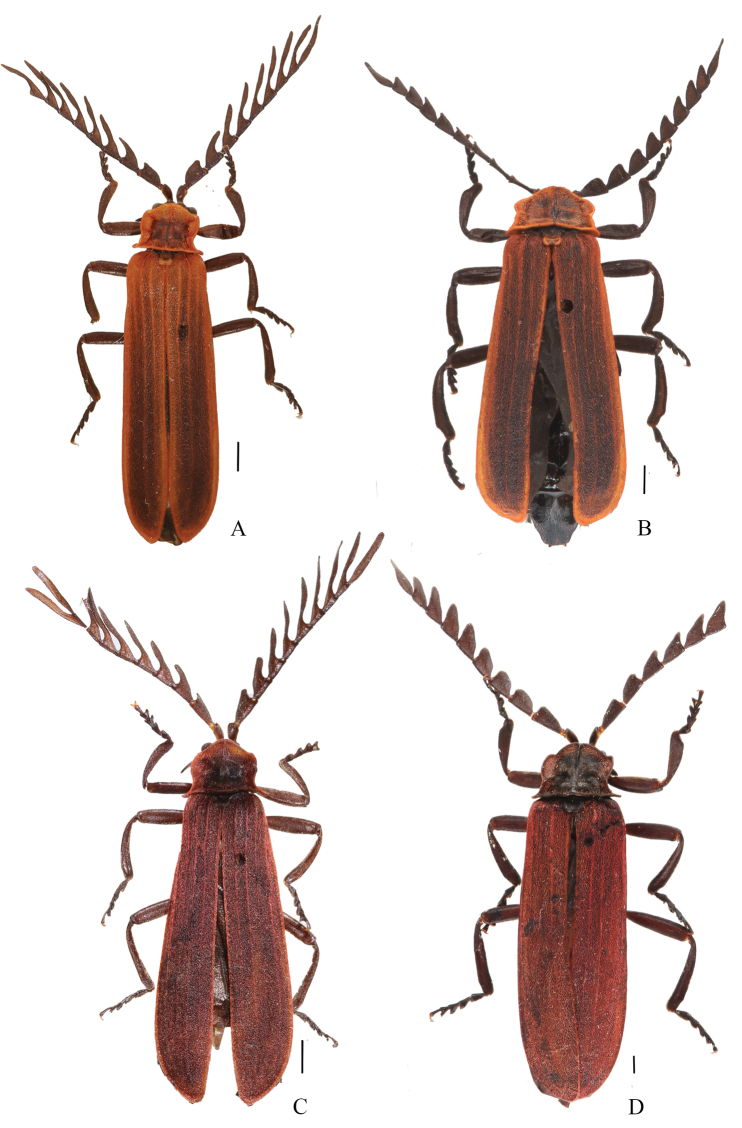
Habitus (dorsal views) of *Macrolycusopacipennis* sp. nov. (**A, B**) and *M.curtus* sp. nov. (**C, D**) **A, C** males **B, D** females. Scale bars: 1.0 mm.

This species differs from all others of the *M.ligulatus* species-group in the elytra darkened at costal intervals (Fig. 7A), while never darkened in others; basal part of phallus progressively widened towards middle in dorsal and ventral views (Fig. 6G, H), while narrowed towards middle or subparallel-sided in others.

##### Etymology.

The specific name is derived from the Latin *opacus* (darkened, obscure) + *penna* (feather), referring to its elytra darkened at costal intervals.

##### Type material.

***Holotype***. China: ♂ (MHBU), Ningxia, Jingyuan, Wanghuanan, 3–4.vii.2009, leg. G. D. Ren & Y. B. Ba. ***Paratype*.** 1 ♀ (MHBU), same data as holotype.

##### Description.

**Male** (Fig. 7A). Length 12.1 mm, width at humeri 2.7 mm.

Body black. Pronotum red with a square black patch in the center of the disc, elytra red and darkened at costal intervals, and scutellum red. Surface covered with decumbent red pubescence (Fig. 7A).

Eyes small, interocular distance about twice greater than eye diameter. Antennae flabellate, overlapping basal 2/3 length of elytra when inclined. Antennomere III with minute lamella, 0.6 times as long as joint itself and apically obtuse, IV–XI lamellate, lamellae pointed at apices; lamella of IX longest, 2.9 times longer than joint itself (Fig. 7A).

Pronotum square, 1.2 times wider than long. Anterior margin widely rounded, and feebly projecting anteriad, lateral margins sinuate and posterior margin straight; anterior angles rounded, posterior angles sharp and moderately projected. Scutellum trapezoidal, emarginate at apex (Fig. 7A).

Elytra 3.7 times longer than humeral width. Costae I and II as strong as IV, and III visible only basally (Fig. 7A).

Phallus slender (Fig. 6G–I), basal part progressively widened towards middle in dorsal and ventral views (Fig. 6G, H), subapical part strongly and asymmetrically inflated laterally, about 2.2 times as wide as basal part, with an oval ventral-cavity, apical part nearly parallel-sided, apex with a deep V-shaped notch, about 0.39 times as wide as subapical part; basal 1/4 part feebly curved ventrally in lateral view (Fig. 6I), subapical part feebly inflated ventrally, apical part moderately expanded ventrally, apex with a tapered lamella.

**Female** (Fig. 7B). Similar to male, but larger in body size. Length 13.0 mm, width at humeri 3.4 mm. Antennae serrate and shorter, overlapping basal 1/3 length of elytra when inclined. Pronotum 1.4 times wider than long. Elytra 3.0 times longer than humeral width, uncovering abdominal tergite VIII.

##### Distribution

**(Fig. 2).** China (Ningxia).

#### ﻿ Macrolycus curtus

Taxon classificationAnimaliaColeopteraLycidae

Y. Yang, Liu & X. Yang sp. nov.

F151EABD-92CE-52CC-AB02-EBC3BECAB4FD

https://zoobank.org/D315E9DC-6731-4A7A-9578-927ED25DAD3D

Figs 6J–L , 7C, D

##### Diagnosis.

The species resembles *M.costus* sp. nov. in the short apical part (1/10 length) and fusiform ventral-cavity of phallus, but differs in the weak elytral costa III (Fig. 7C) (strong in *M.costus* sp. nov.; Fig. 5C); phallus relatively stout and curved laterally at basal 1/4 portion in dorsal and ventral views (Fig. 6J–K) (slender and almost straight at basal part in dorsal and ventral views in *M.costus* sp. nov. (Fig. 6D–F).

##### Etymology.

The specific name is derived from the Latin *curtus* (short), referring to the short apical part of its phallus.

##### Type material.

***Holotype*.** China: ♂ (IZAS), Gansu, Kangxian, Qinghe Forestry, 1400 m, 8.vii.1999, leg. J. Yao. ***Paratype***. 1 ♀ (IZAS), same data as holotype.

##### Description.

**Male** (Fig. 7C). Length 11.5 mm, width at humeri 2.4 mm.

Body black. Pronotum dark red with a square black patch in the center of the disc, elytra and scutellum dark red. Surface covered with decumbent red pubescence (Fig. 7C).

Eyes small, interocular distance about 1.9 times greater than eye diameter. Antennae flabellate, overlapping basal 2/3 length of elytra when inclined. Antennomeres III–XI lamellate, lamellae of III and IV apically rounded and V–XI pointed at apices, lamella of III 0.6 times as long as joint itself, lamella of IX longest, 3.4 times longer than joint itself (Fig. 7C).

Pronotum square, 1.2 times wider than long. Anterior margin widely rounded, and feebly projecting anteriad, lateral margins sinuate and posterior margin almost straight; anterior angles rounded, posterior angles sharp and moderately projected. Scutellum trapezoidal, feebly emarginate at apex (Fig. 7C).

Elytra 4.0 times longer than humeral width. Costae I and II as strong as IV, and III visible only basally (Fig. 7C).

Phallus slender (Fig. 6J–L), basal part parallel-sided and curved laterally at basal 1/4 portion in dorsal and ventral views (Fig. 6J, K), subapical part moderately and asymmetrically inflated laterally, about 1.9 times as wide as basal part, with a fusiform ventral-cavity, apical part constricted distad, apex with a shallow V-shaped notch, about 0.47 times as wide as subapical part; basal 1/3 part moderately curved ventrally in lateral view (Fig. 6L), subapical part flat ventrally, apical part short and moderately expanded ventrally, with a tapered lamella.

**Female** (Fig. 7D). Similar to male, but larger in body size. Length 21.2 mm, width at humeri 5.4 mm. Antennae serrate and shorter, overlapping elytral mid-length when inclined. Pronotum 1.3 times wider than long. Elytra 3.3 times longer than humeral width.

##### Distribution

**(Fig. 2).** China (Gansu).

## ﻿Discussion

Li et al. (2015) divided the genus *Macrolycus* into nine species-groups based on a molecular phylogeny. The species-groups are generally defined by the shapes of apical part of the phallus and form of its attached structure. The *M.ligulatus* species-group can be distinguished from others by the phallus usually expanded ventrodistally, and present with a U- or V-shaped notch and a tongue-like lamella at the apex (Li et al. 2015). However, some species do not match the diagnosis very well, such as *M.chapaensis* and *M.extrusus*, whose apical parts of the phallus are expanded both ventrally and dorsally, similar to most species of the *M.murzini* species-group. Even more puzzling, some species were originally assigned to the *M.ligulatus* species-group, such as *M.parvus*, *M.bocakorum* and *M.notaticollis* (Li et al. 2015), but the apical parts of their phallus are feebly constricted distad in lateral view, corresponding with the diagnosis of the *M.venustus* species-group (Li et al. 2015). The morphological similarity between the *ligulatus* species-group and the *M.venustus* species-group has been noted by Li et al. (2015), but they were treated as separate groups because they belonged to different clades (although with lower supporting values) recovered in the molecular phylogeny. These individual species were considered to be a result of convergent evolution (Li et al. 2015). In this case, it is difficult to assign a species to the *M.ligulatus* species-group, *M.murzini* species-group or *M.murzini* species-group, if no molecular data is available. More samples or data are required to clarify the classification within *Macrolycus* in the future, which is beyond the scope of this study.

Nevertheless, the five new species discovered in the present study conform very well to the diagnosis of the *M.ligulatus* species-group and can be distinguished from others in the following key.

### ﻿Key to world species of *Macrolycusligulatus* species-group

**Table:** 

1	Apical part of phallus expanded ventrally or both ventrally and dorsally in lateral view (e.g., Figs 4C, F, 6C, F, I, L; Kazantsev 1993: fig. 11; 2001: fig. 22; 2013: fig. 29; Li et al. 2012: fig. 33; 2015: fig. 28)	**2**
–	Apical part of phallus subparallel-sided or feebly constricted distad in lateral view (e.g., Kazantsev 2001: fig. 15; Li et al. 2015: fig. 30; Li 2015: fig. 11Q)	**11**
2	Apical part of phallus expanded both dorsally and ventrally in lateral view (e.g., Kazantsev 2013: fig. 29; Li et al. 2012: fig. 31)	**3**
–	Apical part of phallus expanded only ventrally in lateral view (e.g., Figs 4C, F, 6C, F, I, L; Kazantsev 1993: fig. 11; 2001: fig. 22; Li et al. 2015: fig. 28)	**4**
3	Apical part of phallus abruptly constricted at apical 1/10 portion then expanded distad in ventral view (Kazantsev 2013: fig. 28)	***M.chapaensis* Kazantsev, 2013**
–	Apical part of phallus nearly parallel-sided in ventral view (Li et al. 2012: fig. 32)	***M.extrusus* Li, Bocak & Pang, 2012**
4	Antennomere III lamellate in male (e.g., Figs 3B, 5C, 7A, C; Li et al. 2015: fig. 9)	**5**
–	Antennomere III triangular in male (e.g., Fig. 5A; Kazantsev 1993: fig. 13; Li et al. 2012: fig. 48)	**9**
5	Lamella of antennomere III as long as joint itself (Fig. 7C)	***M.curtus* sp. nov.**
–	Lamella of antennomere III at most 0.8 times as long as joint itself (e.g., Figs 3B, 5C, 7A; Li et al. 2015: fig. 9)	**6**
6	Elytral costa III extremely strong (Fig. 5C); basal part of phallus relatively slender and parallel-sided in ventral view (Fig. 6F)	***M.costus* sp. nov.**
–	Elytral costa III weak (e.g., Figs 3B, 7A; Li et al. 2015: fig. 9); basal part of phallus relatively stout or not parallel-sided in ventral view (e.g., Figs 4E, 6H; Li et al. 2015: fig. 29)	**7**
7	Antennae shorter in males, at most extending to elytral mid-length when inclined (Li et al. 2015: fig. 9)	***M.guangxiensis* Li, Bocak & Pang, 2015**
–	Antennae longer in males, at least reaching apical 2/3 length of elytra when inclined (e.g., Figs 5A, 7A)	**8**
8	Elytra uniformly dark red (Fig. 3B); basal part of phallus feebly narrowed towards middle in dorsal and ventral views (Fig. 4D, E)	***M.expansus* sp. nov.**
–	Elytra darkened at costal intervals (Fig. 7A); basal part of phallus progressively widened towards middle in dorsal and ventral views (Fig. 6G, H)	***M.opacipennis* sp. nov.**
9	Lamellae of antennomeres III and IV obtuse at apices (Fig. 5A)	***M.quartus* sp. nov.**
–	Lamellae of antennomeres III and IV acute at apices (e.g., Kazantsev 1993: fig. 13; Li et al. 2012: fig. 48)	**10**
10	Phallus curved ventrally at basal 1/4 portion in lateral view, apical part short, about 1/12 length of phallus (Kazantsev 1993: fig. 12)	***M.praecellens* Kazantsev, 1993**
–	Phallus curved ventrally at basal 1/3 portion in lateral view, apical part longer, about 1/6 length of phallus (Li et al. 2012: fig. 33)	***M.ligulatus* Li, Bocak & Pang, 2012**
11	Body relatively small, less than 8.0 mm in length; interocular distance 2.1 times greater than eye diameter (Li et al. 2015: fig. 10)	***M.parvus* Li, Bocak & Pang, 2015**
–	Body relatively large, more than 10.0 mm in length; interocular distance at most 1.6 times greater than eye diameter	**12**
12	Basal part of phallus expanded in lateral view (Kazantsev 2001: fig. 15)	***M.bocakorum* Kazantsev, 2001**
–	Basal part of phallus never expanded in lateral view (Li 2015: fig. 11Q)	***M.notaticollis* Pic, 1935**

## Supplementary Material

XML Treatment for Macrolycus guangxiensis

XML Treatment for Macrolycus expansus

XML Treatment for Macrolycus quartus

XML Treatment for Macrolycus costus

XML Treatment for Macrolycus opacipennis

XML Treatment for Macrolycus curtus

## ﻿Additional information

### Author ORCIDs

Ruolan Du https://orcid.org/0009-0003-8774-4001

Yuxia Yang https://orcid.org/0000-0002-3118-6659

Xingke Yang https://orcid.org/0000-0003-3676-6828

Haoyu Liu https://orcid.org/0000-0003-1383-5560

### Data availability

All of the data that support the findings of this study are available in the main text.

## References

[B1] Du RL Yang YX Yang XK Liu HY (2024) A taxonomic study on the nominate subgenus Macrolycus Waterhouse, 1878 from China (Coleoptera, Lycidae). Zootaxa 5424(3): 358–366. 10.11646/zootaxa.5424.3.4 38480281

[B2] Kazantsev SV (1993) Lycides nouveaux ou peu connus de l’Indochine (Coleoptera). Bulletin du Muséum National d’Histoire Naturelle 15(4): 49–68.

[B3] Kazantsev SV (2001) *Macrolycus* Waterhouse, 1878 (Coleoptera: Lycidae) of continental China. Elytron 14: 99–109.

[B4] Kazantsev SV (2002) New and little known species of Lycidae (Coleoptera) from China. Russian Entomological Journal 11: 253–263.

[B5] Kazantsev SV (2005) Morphology of Lycidae with some considerations on evolution of the Coleoptera. Elytron 19: 49–226.

[B6] Kazantsev SV (2013) New net-winged beetles (Coleoptera: Lycidae) from Indochina, with synonymic and taxonomic notes. Kavkazskij Entomologiceskij Bjulleten = Caucasian Entomological Bulletin 9(2): 247–252. 10.23885/1814-3326-2013-9-2-247-252

[B7] Kusy D Motyka M Bocek M Masek M Bocak L (2019) Phylogenomic analysis resolves the relationships among net-winged beetles (Coleoptera: Lycidae) and reveals the parallel evolution of morphological traits. Systematic Entomology 44(4): 911–925. 10.1111/syen.12363

[B8] Li Y (2015) Molecular phylogeny and taxonomy of the tribes Macrolycini and Lyponiini (Coleoptera: Lycidae). Masters thesis, Sun Yat-Sen University, Guangzhou, China (unpublished).

[B9] Li Y Bocak L Pang H (2012) New species of *Macrolycus* Waterhouse, 1878 from China and Laos, with a checklist of the genus (Coleoptera: Lycidae). Zootaxa 3232(1): 44–61. 10.11646/zootaxa.3232.1.2

[B10] Li Y Bocak L Pang H (2015) Molecular phylogeny of *Macrolycus* (Coleoptera: Lycidae) with description of new species from China. Entomological Science 18(3): 319–329. 10.1111/ens.12133

[B11] Liu HY Du RL Zhao W Yang XK Yang YX (2023) A morphometric approach to the comparative morphology of aedeagi shapes in net-winged beetles: A case study on the *Macrolycusdotatus* species group (Coleoptera: Lycidae). Arthropod Systematics & Phylogeny 81: 897–916.

[B12] Nakane T (1969) Lycidae (Insecta: Coleoptera). Fauna Japonica. Academic Press of Japan, Tokyo, [viii +] 224 pp. [8 pls]

[B13] Nakane T (1994) Revisional notes on the species of the genus *Macrolycus* Waterhouse in Japan (Insecta, Coleoptera, Lycidae). Biochemical Society Transactions 44: 3–9.

